# Novel *MORC2* variants in Charcot–Marie–Tooth disease type 2Z: genetic and functional insights

**DOI:** 10.3389/fmed.2026.1864944

**Published:** 2026-08-12

**Authors:** Rong Tao, Ten Zhang, Fangmei Luo, Yihui Liu, Liangliang Fan, Xuan Wu, Yuxing Liu

**Affiliations:** 1 Center for Medical Research, Pingshan District Central Hospital of Shenzhen, Shenzhen, Guangdong, China; 2 Department of Neurology, Affiliated Hospital of Yangzhou University, Yangzhou, China; 3 Department of Cell Biology, The School of Life Sciences, Central South University, Changsha, China; 4 Laboratory Center, Guangxi Key Laboratory of Metabolic Reprogramming and Intelligent Medical Engineering for Chronic Diseases, The Second Affiliated Hospital of Guilin Medical University, Guilin, China; 5 Laboratory Center, Guangxi Health Commission Key Laboratory of Glucose and Lipid Metabolism Disorders, The Second Affiliated Hospital of Guilin Medical University, Guilin, China

**Keywords:** axonal neuropathy, CMT2Z, *MORC2*, variant, WES

## Abstract

**Background:**

Charcot–Marie–Tooth disease type 2Z (CMT2Z) is an uncommon inherited condition characterized by autosomal dominant inheritance. It is marked by axonal neuropathy, leading to progressive muscle weakness, cramps, and sensory loss. *MORC2* is one of the candidate pathogenic genes for CMT2Z.

**Methods:**

Here, we enrolled two families affected by Charcot–Marie–Tooth (CMT) disease, with both families having probands who exhibit distal lower limb weakness. Whole-exome sequencing (WES) was used to explore the genetic lesion.

**Results:**

Two heterozygous variants (NM_001303256, c.34G > C;p.A12P; NM_001303256, c.848G > A;p.R283H) of *MORC2* gene were found. The c.848G > A;p.R283H variant is rare, and the c.34G > C;p.A12P variant has not been reported before. Functional studies revealed that the c.848G > A;p.R283H variant resulted in reduced transcription of the *MORC2* gene. The c.34G > C;p.A12P variant did not impact *MORC2* gene transcription, yet it influenced the protein’s half-life and stability. Based on genetic analysis, two probands were subsequently diagnosed with CMT2Z.

**Conclusion:**

Our research contributed to the genetic assessment and guidance for these families, reinforcing the notion that *MORC2* is a potential causative gene for CMT2Z.

## Introduction

Charcot–Marie–Tooth (CMT) disease encompasses a group of primary genetic neuropathies characterized by both sensory and motor impairments (1, 2). Recent population-based studies have indicated that reported prevalence rates vary from 1 in 10,000 to 1 in 5,000 among primarily European populations (3). Progress in genetic diagnosis has unveiled extensive genetic diversity within CMT, with over 100 genes identified to date harboring causative mutations (4). However, despite these advancements, a significant proportion of patients clinically diagnosed with CMT remain genetically undetermined, suggesting the presence of unidentified genetic mutations or genomic rearrangements (5). This challenge is particularly pronounced among patients diagnosed with CMT2, where over 60% lack a definitive genetic diagnosis (6).

CMT2 is an axonal form of CMT disease characterized by peripheral axon degeneration, resulting in distal motor dysfunction. As outlined in a recent comprehensive review, at least a dozen genetically distinct CMT2 subtypes have been characterized (7). One such subtype is Charcot–Marie–Tooth disease type 2Z (CMT2Z; OMIM: 616688), which is caused by variants in the Microrchidia CW-type zinc finger protein 2 (*MORC2*) gene (8, 9). Typically, the initial symptoms manifest as cramps in the lower limbs in childhood or early adulthood, eventually evolving into characteristic CMT features such as weakness in the legs and hands, sensory deficits, absent reflexes, and pes cavus. In some older individuals, atypical presentations such as the scapuloperoneal phenotype may emerge, characterized by significant asymmetrical weakness in proximal muscles, particularly those of the pelvic and shoulder girdles (10). CMT2Z is a rare genetic disorder, with fewer than 100 reported cases worldwide. Despite this rarity, more than 20 pathogenic or likely pathogenic variants of *MORC2* have been identified to date, all of which are missense mutations. Recent studies have revealed a prevalence of *MORC2* mutations of 2.1% in Chinese CMT2 patients and 2.7% in Japanese CMT2 patients (11–13).

In this study, we documented two Chinese families exhibiting classic CMT symptoms. Using whole-exome sequencing (WES), we discovered two heterozygous variants in the *MORC2* gene and investigated their impact on protein function through functional experiments.

## Methods

### Subjects

This study enrolled two Chinese families with characteristic CMT symptoms, and blood samples were obtained from every family member. The Ethics Committee of the Affiliated Hospital of Yangzhou University (Yangzhou, China) approved this research. All participants in the study provided their written consent after being fully informed.

### WES

Genomic DNA was extracted from peripheral blood lymphocytes of all using the PureLink™ Genomic DNA Mini Kit (Invitrogen). We selected DNA samples from the probands of two family lineages for WES, with services provided by the Novogene Bioinformatics Institute (Beijing, China). We utilized Agilent SureSelect Human All Exon V6 kits for exome enrichment and performed next-generation sequencing using an Illumina HiSeq X-10 platform, as detailed in our previous studies (14).

### Data filtering

Data filtering adhered to the methods outlined in our earlier research (15, 16). The process generally involved the following steps: (a) selected exonic and splice variants; excluded intronic, intergenic, UTRs, and synonymous SNVs; (b) ACMG-classified pathogenic/likely pathogenic and VUS variants were considered; (c) excluded common variants based on 1000G, dbSNP144, YH, gnomAD v2.1.1, and Berry Genomics exome DB; (d) bioinformatics tools (MutationTaster, PolyPhen-2 v2.2.8, SIFT v6.2.1, MobiDetails) predicted variant impacts; (e) screened variants via OMIM, HGMD, and literature review; and (f) conducted Sanger sequencing and co-segregation analysis on family DNA samples.

### Sanger sequencing

Sanger sequencing confirmed all filtered variants in the patients, with primer sequences detailed in Supplementary Table S1. The polymerase chain reaction (PCR) product sequences were analyzed using an ABI 3100 Genetic Analyzer (ABI, Foster City, CA, United States).

### Nerve conduction velocity assays

NCV assays were conducted to quantitatively analyze the potential induced in the muscles innervated by the nerve stimulated at a specific intensity of pulsed electricity, including measurements of both motor and sensory conduction velocities. These assays were performed on patients at the Affiliated Hospital of Yangzhou University.

### Electromyogram assays

The EMG assay was conducted to quantitatively analyze muscle function by measuring the duration and amplitude of motor unit potentials. The EMG assay was performed on patients at the Affiliated Hospital of Yangzhou University.

### Bioinformatics analysis

The necessary bioinformatics analyses were conducted, including conservation analysis using ConSurf Server, tolerance analysis with MetaDome, and mutant modeling via SWISS-MODEL, as per the methodologies detailed in our previous studies (15, 16).

### Cell lines and key reagents

The human embryonic kidney 293 T (HEK293T) cell line was purchased from the Cell Bank of the Shanghai Institutes for Biological Sciences (Shanghai, China) and was cultured under standard conditions (17). The antibodies of Flag (80010-1-RR) and *β*-actin (20536-1-AP) were purchased from Proteintech. Cycloheximide (CHX, S7418) was purchased from Selleckchem.

### Plasmid construction and transfection

The *MORC2* eukaryotic expression vector with complete coding frame (pcDNA3.1-*MORC2*-flag, WT-*MORC2*) was designed. The c.34G > C-*MORC2* and c.848G > A-*MORC2* missense variants were engineered into the vector above by using a Takara MutanBEST Kit (Takara) (Supplementary Figures S1A,B). HEK293T cells were transiently transfected with pcDNA3.1-*MORC2*-flag (wild type and/or mutant) using a Lipofectamine™ 2000 CD Transfection Reagent (Thermo Fisher Scientific) following the manufacturer’s instructions.

To determine protein half-lives, CHX at a concentration of 100 μg/mL was added to the cell culture post-transfection. Following the commencement of the treatment, cells were collected at specific time points, and protein expression was quantified using Western blot (WB) techniques.

### WB analyses

Equal amounts of protein lysates were separated by SDS-PAGE and visualized by Western blotting using the antibodies specified above. The relative intensities of the spots were determined by the iBright Gel imaging system (Thermo Fisher Scientific).

### RT-PCR and real-time PCR

Total RNA was isolated using an RNA isolation kit and then reverse transcribed into cDNA using the RevertAid First Strand cDNA Synthesis Kit. qPCR was implemented using the SYBR GREEN PCR Kit (Toyobo). The qPCR primer sequences are listed in Supplementary Table S1. The 2^(−△△Ct)^ method was used to compare the RNA levels of each group.

### Statistical analysis

All functional experiments were independently performed at least three times (biological replicates, *n* = 3). The results are presented as mean ± standard deviation. Statistical analysis was conducted using a paired Student’s *t*-test, and a *p*-value of ≤0.05 was considered statistically significant.

## Results

### Clinical features

**Figure 1:**
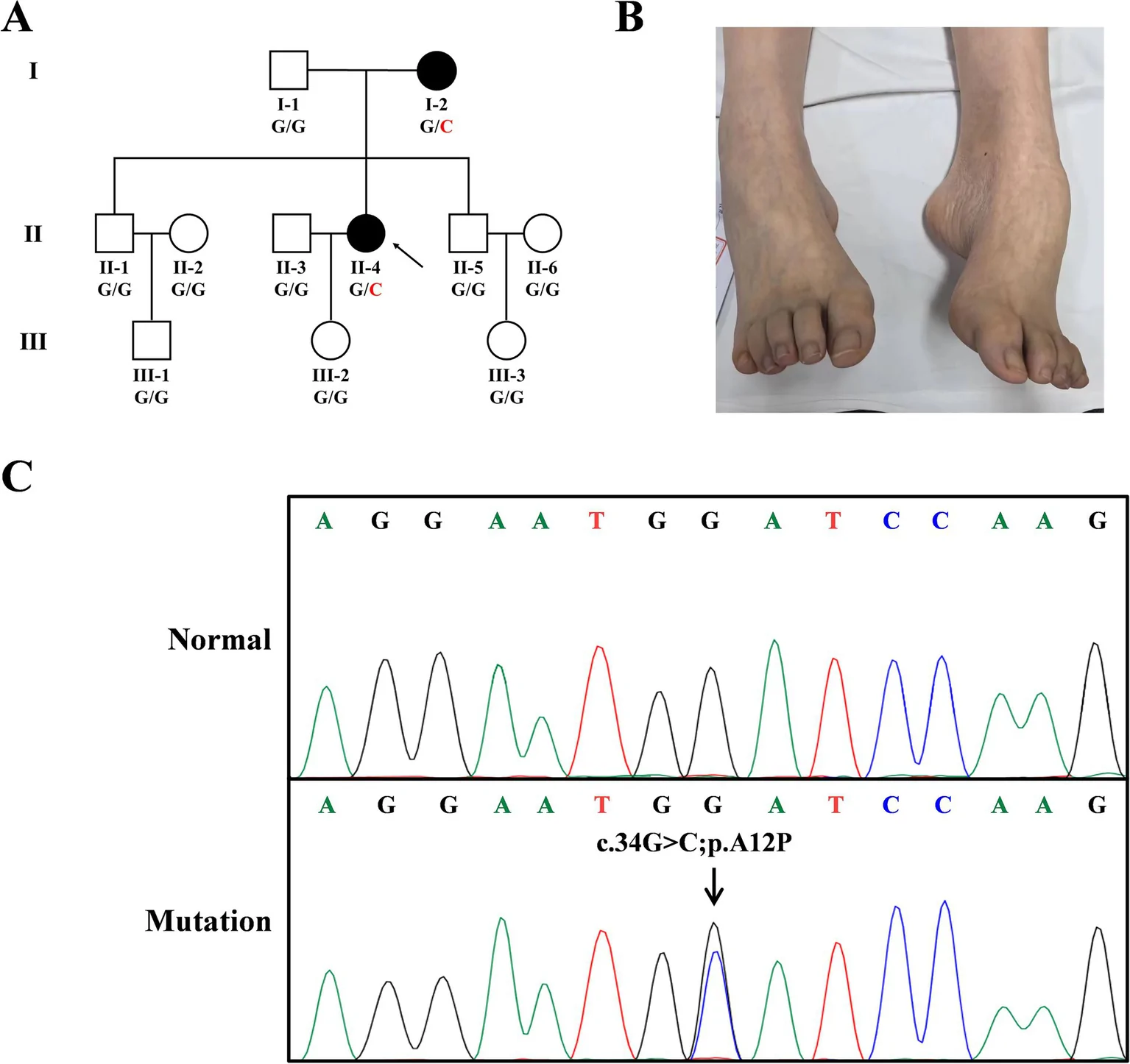
**(A)** Pedigrees of family 1 with segregation analysis. Square denotes a male family member, circle represents a female family member, slashed symbol indicates a deceased family member, fully shaded symbol shows a patient with CMT, and open symbol represents a member with no symptoms. **(B)** Photograph showing pes cavus. **(C)** Sanger DNA sequencing chromatogram demonstrates the heterozygous variant (c.34G > C;p.A12P) in the *MORC2* gene.

The proband 1 was a 35-year-old woman who was hospitalized for distal lower limb weakness, characterized by a propensity to fall while walking, and progressive weakness of both lower extremities at the age of 27 (Figure 1A). The proband was born following a routine pregnancy to a mother who did not encounter any evident complications during labor or the neonatal period. As her condition worsened, she encountered challenges in climbing stairs and performing squats. Physical examination showed the proband to have a thin body, lower limbs with significant deformities, and high-arched feet (Figure 1B). The proband 1’s speech is not smooth, both pupils are equal in size and reactive to light, facial muscles are symmetrically aligned, and the tongue is midline with no signs of atrophy. The strength in both lower limbs is rated at grade 4-. The limbs exhibit reduced muscle tone, with absent tendon reflexes, and there are no signs of bilateral pathological or meningeal irritation. The score of the Montreal Cognitive Assessment (MCA) of her is 18, indicating significant impairments in memory, arithmetic, and executive functioning. The NCV of the proband 1 showed that the sensory nerve action potentials (SNAP) of the upper and lower limbs were not elicited, the motor nerve conduction velocities (MNCV) of the upper and lower limbs were normal, and the compound muscle action potential (CMAP) amplitudes were decreased in some muscles (Tables 1, 2). EMG revealed no spontaneous electrical activity in the muscles of the upper and lower limbs examined, with large motor unit potentials (MUPs) and diminished recruitment (Table 3). Consequently, the initial diagnosis for the proband was CMT. Family history investigation revealed the proband 1’s mother (I-2) also presented symptoms of similar decreased muscle strength and thinness, while other members of the family did not show any related symptoms.

**Table 1:** Motor nerve conduction velocity measurement results of the proband 1.

Nerve	Latency (ms)	Amplitude (mv)	CV (m/s)
L-Median motor nerve			
Wrist-Elbow	3.2	7.4	54.5
R-Median motor nerve			
Wrist-Elbow	2.5	13.1	55.0
L-Ulnar motor nerve			
Wrist-5cmBl.Elbow	NA	NA	NA
5cmBl.Elbow-5cmAb.Elbow	NA	NA	NA
R-Ulnar motor nerve			
Wrist-5cmBl.Elbow	2.5	10.7	58.1
5cmBl.Elbow-5cmAb.Elbow	5.1	10.2	50.1
L-Peroneal motor nerve			
Ab.LM-FH	NA	NA	NA
R-Peroneal motor nerve			
Ab.LM-FH	2.5	5.8	44.4
L-Tibial motor nerve			
Bl.MM-PF	3.1	4.9	42.9
R-Tibial motor nerve			
Bl.MM-PF	2.9	1.9	45.7

LM, lateral malleolus; FH, fibular head; MM, medial malleolus; PF, popliteal fossa; CV, conduction velocity; L, left; R, right; NA, not available.

**Table 2:** Sensory nerve conduction velocity measurement results of the proband 1.

Nerve	Latency (ms)	Amplitude (uv)	Distance (mm)	CV (m/s)
L-Ulnar sensory nerve				
Fifth Digit-Wrist	NP	NA	NA	NA
R-Ulnar sensory nerve				
Fifth Digit-Wrist	NP	NA	NA	NA
L-Median sensory nerve				
Third Digit-Wrist	NP	NA	NA	NA
R-Median sensory nerve				
Third Digit-Wrist	NP	NA	NA	NA
L-Superficial radial nerve				
7.5cmAb.Wrist-TE	NA	NA	NA	NA
R-Superficial radial nerve				
7.5cmAb.Wrist-TE	NP	NA	NA	NA
L-Superficial peroneal sensory nerve				
Bl.MM-PF	NP	NA	NA	NA
R-Superficial peroneal sensory nerve				
Bl.MM-PF	NP	NA	NA	NA
L-Sural sensory nerve				
12cmAb.HC-Bl.LM	NP	NA	NA	NA
R-Sural sensory nerve				
12cmAb.HC-Bl.LM	NP	NA	NA	NA

TE, thenar eminence; MM, medial malleolus; PF, popliteal fossa; HC, heel counter; LM, lateral malleolus; CV, conduction velocity; L, left; R, right; NA, not available; NP, no potential.

**Table 3:** Needle electromyography results of the proband 1.

Examined muscle	Insertion activity	Spontaneous activity			MUAP					Recruitment
		Fib	PSW	Fas	Dur	Amp	Poly	Irregular waves	Shape	
L-1^st^ Interosseous muscle	Normal	−	−	Normal	Normal	Normal	Increased	Increased	>5mv	Reduced
L-Biceps brachii	Normal	−	−	Normal	Normal	Normal	Increased	None	Normal	Reduced
L-Tibialis anterior	Normal	+	+	Normal	Normal	Normal	None	None	>5mv	Reduced
L-Gastrocnemius (medial)	Normal	+	+	Normal	Normal	Normal	Increased	None	>5mv	Reduced
L-Rectus femoris	Normal	Normal	Normal	Normal	Normal	Normal	Increased	Increased	Normal	Reduced
R-1^st^ Interosseous muscle	Normal	−	−	Normal	Normal	Normal	None	None	>5mv	Reduced
R-Biceps brachii	Normal	−	−	Normal	Normal	Normal	Increased	None	>5mv	Reduced
R-Tibialis anterior	Normal	+	+	Normal	Normal	Normal	Increased	Increased	>5mv	Reduced
R-Gastrocnemius (medial)	Normal	+	+	Normal	Normal	Normal	Increased	Increased	>5mv	Reduced
R-Rectus femoris	Normal	−	−	Normal	Normal	Normal	Increased	increased	>5mv	Reduced

Fib, fibrillation; PSW, positive sharp wave; Fas, fasciculation; MUAP, motor unit action potential; Dur, duration; Amp, amplitude; Poly, polyphasic wave; L, left; R, right.

**Figure 2:**
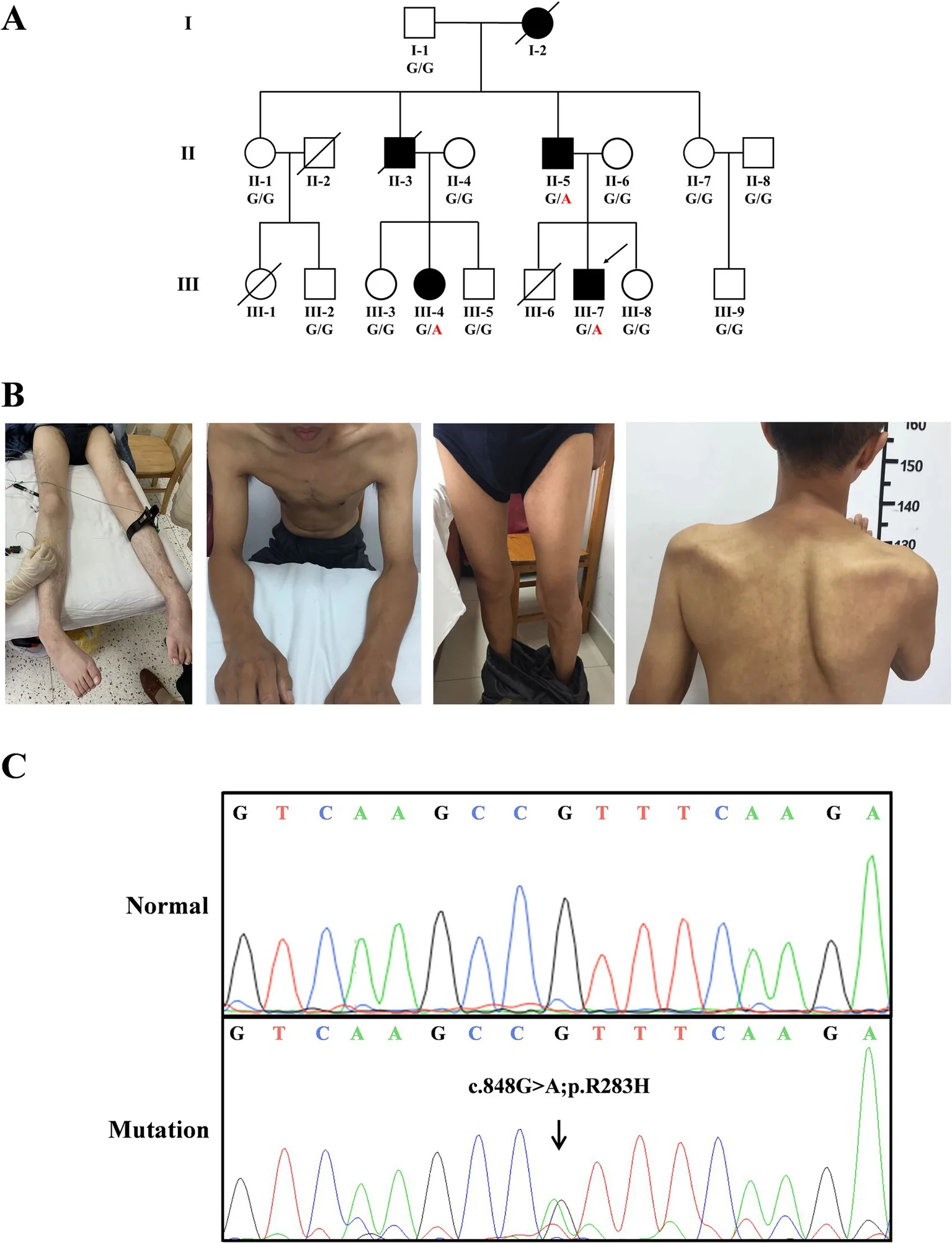
**(A)** Pedigrees of family 2 with segregation analysis. Square denotes a male family member, a circle represents a female family member, a slashed symbol indicates a deceased family member, a fully shaded symbol shows a patient with CMT, and an open symbol represents a member with no symptoms. **(B)** Photograph shows pes cavus, muscle atrophy in the lower and upper limbs, and prominent asymmetric proximal muscle weakness (the scapuloperoneal phenotype). **(C)** Sanger DNA sequencing chromatogram demonstrates the heterozygous variant (c.848G > A/p.R283H) in the *MORC2* gene.

The proband 2 was a 44-year-old man who was hospitalized for muscle weakness (Figure 2A). He presented with decreased muscle strength and thinness (Figure 2B). The NCV results of proband 2 showed dual lateral movement of the median nerve conduction velocity slightly slow with CMAP amplitude mildly decreased, bilateral ulnar nerve conduction velocity slightly slow, reduced SNAP amplitude in the upper extremities of the sensory nerves, and slight extension of motor F-wave latency was detected in some upper limbs (Tables 4, 5). In the EMG assay, the lower limb showed a small amount of spontaneous potential, and in light contraction, the examined muscle showed relatively generous or huge potentials with irregular polyphasic potentials, and the amplitude of hefty contraction was reduced (Table 6). A wide range of neurogenic lesions have been shown by electromyogram assay, which focused on chronic damage and reinnervation change, including lesions in the muscles of the upper and lower extremities, rectus abdominis, masseter, and tongue. His ventricornu cells and cranial nerve motor nuclei may be injured. The electrophysiology of the upper limbs manifested multiple peripheral nerve damage, especially sensory axonal damage. Hence, CMT was the initial diagnosis of the proband 2. The family history investigation revealed that the proband’s father (II-5), deceased grandmother (I-2), deceased uncle (II-3), and one of the uncle’s daughters (III-4) all exhibited similar symptoms of decreased muscle strength and thinness. Other family members showed no such symptoms and were considered normal. In addition, the proband is unmarried. Among the siblings of the same generation, those who are married (III-2, III-3, and III-8) have not yet observed similar symptoms in their offspring.

**Table 4:** Motor nerve conduction velocity measurement results of the proband 2.

Nerve	Latency (ms)	Amplitude (mv)	CV (m/s)
L-Ulnar motor nerve			
Wrist-ADM	2.63	7.10	NA
Bl.Elbow-Wrist	6.27	6.70	54.90
Ab.Elbow-Bl.Elbow	8.94	5.10	44.90
R-Ulnar motor nerve			
Wrist-ADM	2.40	8.10	NA
Bl.Elbow-Wrist	6.56	6.30	52.00
Ab.Elbow-Bl.Elow	9.30	5.80	47.60
R-Radial motor nerve			
Forearm–EIP	2.92	2.20	NA
Ab.Elbow-Forearm	5.44	2.20	59.50
Erb-Ab.Elbow	9.21	2.20	79.60
L-Median motor nerve			
Wrist-APB	2.38	5.20	NA
Elbow-Wrist	7.35	4.70	48.30
R-Median motor nerve			
Wrist-APB	3.38	3.30	NA
Elbow-Wrist	7.94	3.40	49.30
L-Tibial motor nerve			
Malleolus-AH	3.91	8.20	NA
PF-Malleolus	11.50	5.90	49.40
R-Tibial motor nerve			
Malleolus-AH	3.01	9.70	NA
PF-Malleolus	12.10	6.80	41.80
L-Peroneal motor nerve			
Malleolus-EDB	3.75	3.40	NA
Bl.Knee-Malleolus	10.40	2.90	42.10
Ab.Knee-Bl.Knee	13.00	2.90	42.30
R-Peroneal motor nerve			
Malleolus-EDB	3.85	3.60	NA
Bl.Knee-Malleolus	10.40	3.30	45.80
Ab.Knee-Bl.Knee	12.40	3.00	50.0

ADM, abductor digiti minimi; EIP, Extensor indicis proprius; APB, abductor pollicis brevis; PF, popliteal fossa; EDB, extensor digitorum brevis; AH, abductor hallucis; CV, conduction velocity; L, left; R, right; NA, not available.

**Table 5:** Sensory nerve conduction velocity measurement results of the proband 2.

Nerve	Latency (ms)	Amplitude (uv)	Distance (mm)	CV (m/s)
L-Ulnar sensory nerve				
5th Digit-Wrist	2.08	1.61	110.00	52.90
R-Ulnar sensory nerve				
5^th^ Digit-Wrist	2.02	3.80	103.00	51.00
L-Radial sensory nerve				
EPL tendon-Wrist	1.73	10.60	100.00	57.80
R-Radial sensory nerve				
EPL tendon-Wrist	1.94	9.90	115.00	59.30
L-Median sensory nerve				
3rd Digit-Wrist	2.44	4.40	130	53.30
R-Median sensory nerve				
3^rd^ Digit-Wrist	2.27	6.80	135.00	59.50
L-Superficial peroneal sensory nerve				
Malleolus-Dorsum pedis	NA	NP	NA	NA
R-Superficial peroneal sensory nerve				
Malleolus-Dorsum pedis	2.30	11.00	105.00	45.70
L-Sural sensory nerve				
Middle calf-LM	1.78	16.30	95.00	53.40
R-Sural sensory nerve				
Middle calf-LM	1.78	16.50	90.00	50.60

EPL, extensor pollicis longus; LM, lateral malleolus; CV, conduction velocity; L, left; R, right; NA, not available; NP, no potential.

**Table 6:** Needle electromyography results of the proband 2.

Examined muscle	Insertion activity	Spontaneous activity			MUAP					Recruitment
		Fib	PSW	Fas	Dur	Amp	Poly	Irregular waves	Shape	
L-Glossus	Normal	Normal	Normal	Normal	Normal	Normal	Increased	Increased	>5mv	Reduced
L-Masseter	Normal	Normal	Normal	Normal	Normal	Normal	Increased	None	>5mv	Reduced
L-Sternocleidomastoid	Normal	Normal	Normal	Normal	Normal	Normal	None	None	>5mv	Reduced
L-Trapezius	Normal	Normal	Normal	Normal	Normal	Normal	Increased	None	>5mv	Reduced
R-Biceps brachii	Normal	Normal	Normal	Normal	Normal	Normal	Increased	Increased	>5mv	Reduced
R-Dorsal interossei I	Normal	Normal	Normal	Normal	Normal	Normal	None	None	>5mv	Reduced
R-Rectus femoris	Increased	Normal	+	+	Normal	Normal	Increased	None	>5mv	Reduced
R-Gastrocnemius (lateral)	Increased	Normal	+	Normal	Normal	Normal	Increased	Increased	>5mv	Reduced
R-Tibialis anterior	Increased	Normal	+	Normal	Normal	Normal	Increased	Increased	>5mv	Reduced

Fib, fibrillation; PSW, positive sharp wave; Fas, fasciculation; MUAP, motor unit action potential; Dur, duration; Amp, amplitude; Poly, polyphasic wave; L, left; R, right.

### Molecular genetic analysis

WES data for proband 1 provided 8.02 Gb with 97.10% target region coverage and 96.03% coverage at 10 × depth. After alignment and single nucleotide variant calling, 80,197 variants were identified in the proband. By the above-mentioned filtering method, 11 variants were detected (Table 7). After Family overlapping, a missense variant (NM_001303256, c.34G > C;p.A12P) of the *MORC2* gene was identified. Sanger sequencing revealed that the variant (c.34G > C;p.A12P) identified in proband 1 was inherited from her mother (I-2) (Figures 1A,C). Proband 2’s WES produced 7.24 Gb data, with 98.04% target region coverage and 96.93% at 10 × depth. Post-alignment and variant calling, 83,802 variants were identified, and 8 variants were detected after filtering (Table 7). After Family overlapping, another missense variant (c.848G > A;p.R283H) of *MORC2* was indicated and was co-segregated in family 2 (Figures 2A,C).

**Table 7:** Sequencing results of two subjects in this study.

Patient	Gene	Variant	MutationTaster	PolyPhen-2	SIFT	gnomAD	OMIM clinical phenotype	ACMG classification
1	*RNF220*	c.711G > T, p.Q237H	D	D	D	–	AR, leukodystrophy; AR, liver dysfunction; AR, dilated cardiomyopathy	PM2, PP3
	*LMAN2L*	c.601C > G, p.R201G	D	D	D	–	AD, intellectual developmental disorder; AR, intellectual developmental disorder	PM2, PP3
	*BDP1*	c.431G > A, p.R144Q	D	D	D	0.0000096	AR, deafness	PM2, PP3
	*MYO6*	c.1139G > A, p.G380D	D	D	D	–	AD, deafness with hypertrophic cardiomyopathy; AR, deafness	PM2, PP3
	*PRAG1*	c.337 T > C, p.W113R	–	–	–	–	–	PM2
	*DCHS1*	c.3542 T > C, p.V1181A	D	D	D	–	AD, mitral valve prolapse; AR, Van Maldergem syndrome	PM2, PP3
	*ITGA11*	c.3428G > T, p.W1143L	D	D	D	–	–	PM2, PP3
	*TOX3*	c.1720A > G, p.S574G,	D	D	D	–	–	PM2, PP3
	*LRP3*	c.1273G > T, p.G425C	D	D	D	–	–	PM2, PP3
	**MORC2**	c.34G > C, p.A12P	D	D	D	–	AD, Charcot–Marie–Tooth disease, axonal, type 2Z; AD, Developmental delay, impaired growth, dysmorphic facies, and axonal neuropathy	PS3, PM5, PP2, PM2, PP4, PP1
	*SH3KBP1*	c.347C > A, p.T116N	D	D	D	–	XLR, immunodeficiency	PM2, PP3
2	*KIAA1217*	c.1468G > A, p.V490M	D	D	D	–	–	PM1, PM2, PP3
	*PAK6*	c.431A > G, p.Y144C	D	D	D	–	–	PM2
	*B9D1*	c.392A > C, p.Q131P	D	D	D	–	AR, Meckel syndrome 9; AR, Joubert syndrome 27	PM2, PP3
	*OSBPL1A*	c.80C > G, p.P27R	D	D	D	–	–	PM1, PM2, PP3
	*ROMO1*	c.4C > G, p.P2A	D	D	D	–	–	PM2, PP3
	**MORC2**	c.848G > A, p.R283H	D	D	D	0.0000289	AD, Charcot–Marie–Tooth disease, axonal, type 2Z	PS3, PP2, PM1, PM2
	*DNAH11*	c.6046 T > A, p.C2016S	D	D	D	–	AR, Ciliary dyskinesia, primary, 7, with or without situs inversus	PM1, PM2
	*PAG1*	c.268G > A, p.G90R	D	D	D	–	–	PM2, PP3

Bold text, variants identified in this study; D, disease causing; AR, autosomal recessive; AD, autosomal dominant.

### Bioinformatics analysis

**Figure 3:**
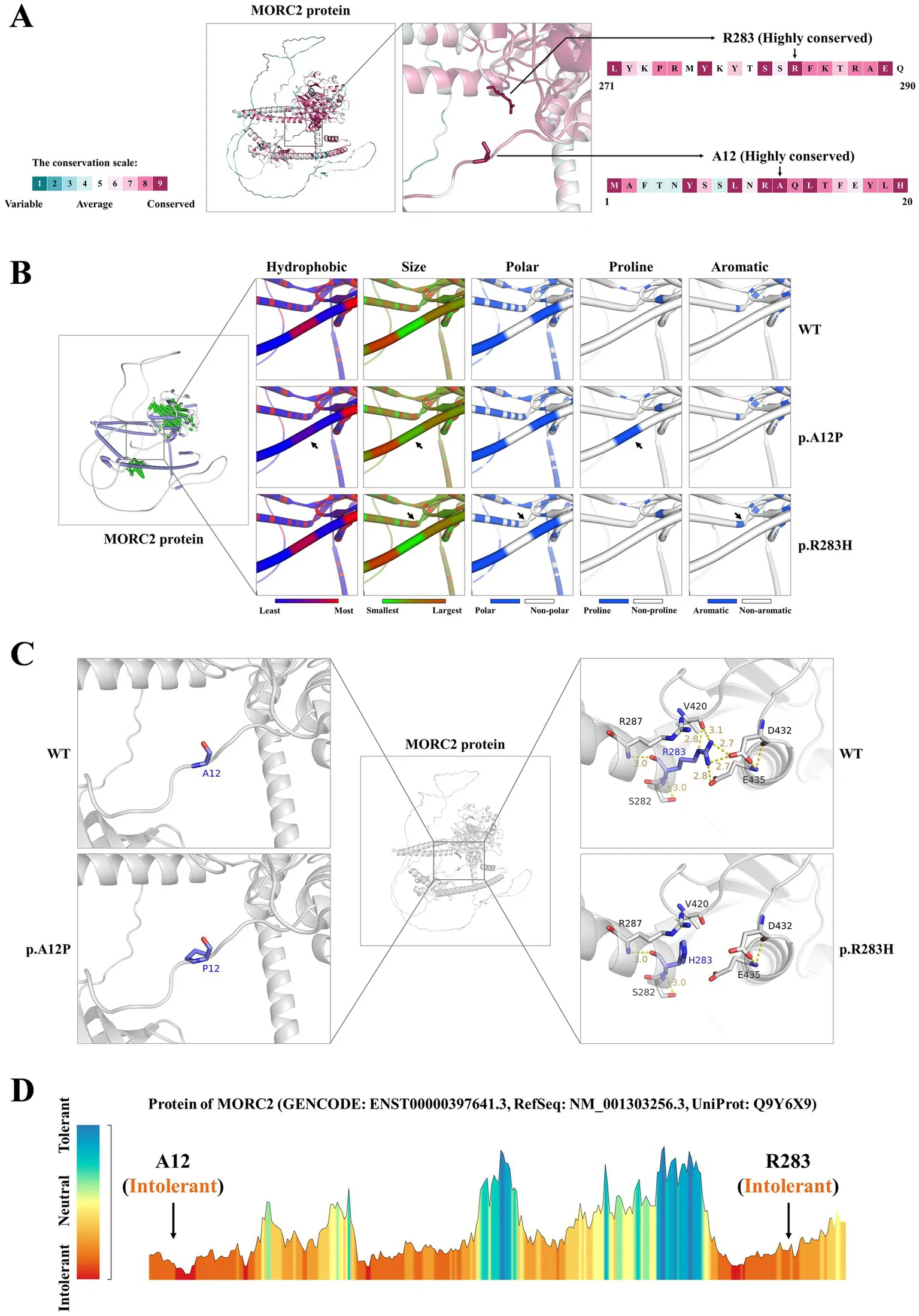
**(A)** Conservation analysis of mutant amino acid sites of *MORC2* was predicted by ConSurf Server software. **(B,C)** Protein models of *MORC2* with or without mutants were predicted using the SWISS-MODEL online software. Black arrows indicate changes caused by mutation, blue words indicate affected amino acids, and yellow words represent hydrogen bond distances. **(D)** The tolerance analysis of mutant amino acid sites in *MORC2* was performed using MetaDome software.

We subsequently conducted bioinformatics assessments to determine the pathogenic potential of these two variants. The ConSurf Server software indicated that both targeted amino acids exhibited high conservation (Figure 3A). SWISS-MODEL software was used to model mutant proteins for the two variants and to visually analyze changes in the physicochemical properties of the proteins. The results showed that the p.A12P variant altered the hydrophobicity, size, and proline fraction at that site of the protein. In contrast, the other variant, p.R283H, affected the size, polarity, and aromatic fraction of the protein (Figure 3B). We examined the effects of the mutations on hydrogen bonding with neighboring amino acids. The findings showed that the A12 residue lacked hydrogen bonding with surrounding residues in the 3D structure, and this remained unchanged post-mutation. In contrast, the R283 residue formed hydrogen bonds with multiple surrounding amino acids, and the variant disrupted most of these hydrogen bonds, potentially causing significant effects on the protein (Figure 3C). Furthermore, MetaDome software analysis shows that two affected amino acid sites have low tolerance to mutations (Figure 3D).

### Functional analysis

**Figure 4:**
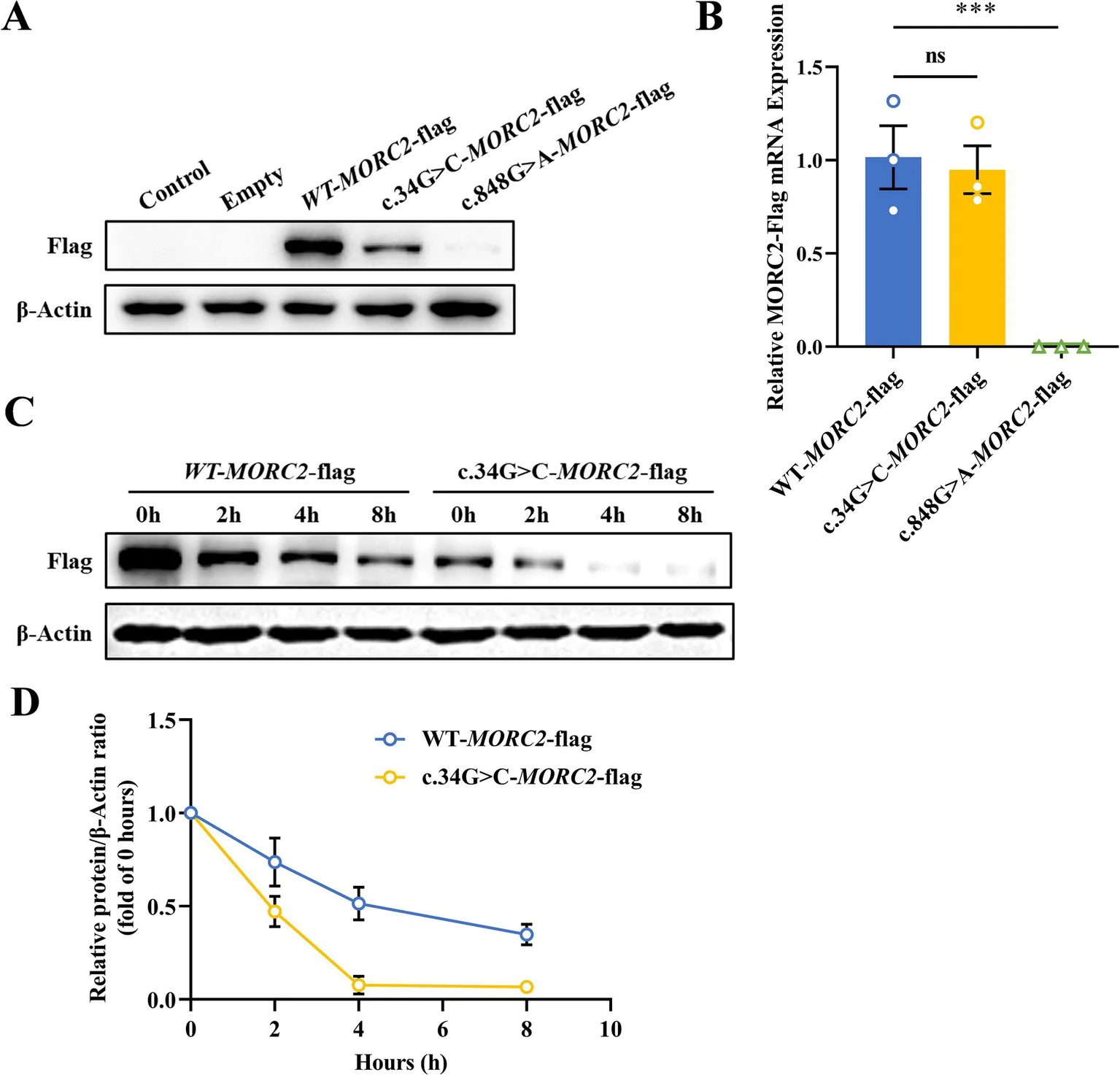
**(A)** WB detected the expression of transfected *MORC2* (Flag) in each group. **(B)** qPCR detected the mRNA levels of transfected *MORC2* (*Flag*) in each group. **(C)** WB detected the degradation rate of transfected *MORC2* (Flag) in WT-‑*MORC2*‑flag and c.34G>C‑-*MORC2*‑flag groups. **(D)** Densitometric quantification of the Western blot bands shown in **(C)**. **p < 0.001, ns represents no significant difference.

We constructed normal *MORC2* plasmids (WT-*MORC2*-flag) and two mutant *MORC2* plasmids separately (c.34G > C-*MORC2*-flag and c.848G > A-*MORC2*-flag), and transfected them into HEK293T cells (Figure 4A). Compared to the WT-*MORC2*-flag group, WB experiments showed that the expression of transfected *MORC2* (Flag) signal was significantly decreased in both mutant *MORC2* plasmid groups (Figure 4B). We examined the mRNA levels of *MORC2* in each group. The results indicated that, compared to the WT-*MORC2*-flag group, there was no significant difference in *MORC2* mRNA levels in the c.34G > C-*MORC2*-flag group, while *MORC2* mRNA levels in the c.848G > A-*MORC2*-flag group were significantly decreased (Figure 4C). Given that the mRNA levels were comparable between the WT-*MORC2*-flag and c.34G > C-*MORC2*-flag groups, we treated the transfected cells with CHX for 36-h post-plasmid transfection. WB analysis indicated that the degradation rate of *MORC2* (Flag) in the c.34G > C-*MORC2*-flag group was faster than in the WT-*MORC2*-flag group (Figure 4D).

## Discussion

In this study, we explored the genetic causes of CMT in two families through whole-exome sequencing (WES). In each family, we identified a different heterozygous variant in the *MORC2* gene, namely c.34G > C;p.A12P in family 1 and c.848G > A;p.R283H in family 2. Both variants co-segregated with the disease within their respective families. According to the ACMG/AMP guidelines, the c.34G > C;p.A12P variant met the criteria PS3, PM5, PP2, PM2, PP4, and PP1, while the c.848G > A;p.R283H variant met PS3, PP2, PM1, and PM2. Both variants were therefore classified as likely pathogenic. Based on these genetic and functional findings, the probands of both families were molecularly diagnosed with CMT2Z.

**Figure 5:**
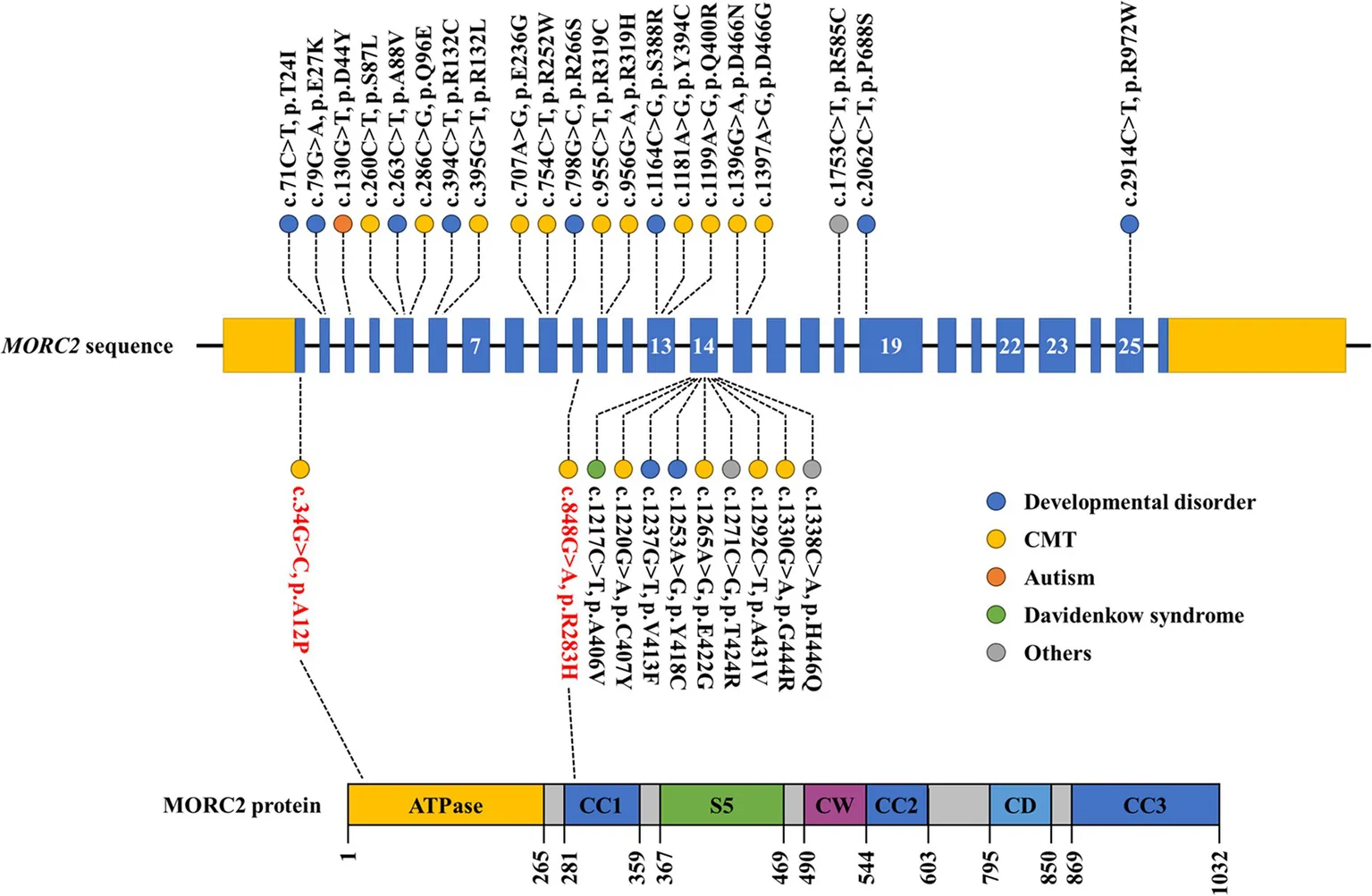
Overview of all reported mutations in the *MOCR2* gene. CMT, Charcot–Marie–Tooth; CC, coiled-coil domain; S5, ribosomal protein S5 domain; CW, CW-type finger domain; CD, Chromo-like domain. Red words indicate mutations were identified in this study.

The *MORC2* gene, mapped to chromosome 22q12.2, has 26 exons and a 5,657-bp open reading frame, and encodes a 1,032-amino acid protein. *MORC2* is a crucial ATP-dependent chromatin remodeling factor involved in all DNA-templated processes in eukaryotes, and its dysregulation is closely associated with a range of human ailments. As a highly conserved nuclear matrix protein, *MORC2* is characterized by the presence of an ATPase domain, a central CW-type zinc finger (CW-ZF) domain, a C-terminal chromo-like domain, and several coiled-coil domains (18, 19). The ATPase region of *MORC2* plays a pivotal role in epigenetic gene regulation and the repair of DNA damage (20). Notably, alterations within this domain of *MORC2* have been linked to the pathogenesis of hereditary CMT (21). The CW-ZF domain acts as a histone recognition module for trimethylation of histone H3 at lysine 4 (H3K4me3) (22). The C-terminal chromo-like domain of *MORC2* is responsible for recognizing methylated peptides on both histones and non-histone proteins, whereas the coiled-coil domains facilitate protein complex formation and molecular recognition (12, 23). In this study, the p.A12P variant is located in the ATPase domain of *MORC2*, potentially affecting its ATPase activity and leading to CMT. The R283H variant is located in the first coiled-coil domain (Figure 5). Protein modeling results showed that this variant, located in an *α*-helix, replaced a linear amino acid with a cyclic amino acid, altering several hydrogen bonds originally formed with surrounding amino acids (Figure 3C). Although the function of the first coiled-coil domain in the *MORC2* protein is currently unknown, several mutations in this domain have already been reported to be associated with CMT (24, 25).

The connection between *MORC2* variants and human disease was identified relatively recently, primarily through studies of neuropathy (26). In 2016, *MORC2* was first reported as a causative gene for autosomal dominant CMT in Spanish families (27). To date, only 31 mutations in the *MORC2* gene have been reported in the literature, all of which are missense. Of these, 50% are associated with symptoms of CMT, while the remaining mutations are linked to conditions such as developmental abnormalities, autism, and Davidenkow syndrome (Figure 5). Although deletions and nonsense variants appear in some databases, they currently lack clinical evidence of pathogenicity. In this study, we reported two different *MORC2* variants in two separate CMT Chinese families from China. The p.A12P variant found in family 1 is unprecedented in the literature, making it a novel mutation. The p.R283H (ClinVar variation ID: 951841) variant identified in family 2 was first reported by Albulym et al. as a non-segregating *MORC2* variant (28). In their study, this variant did not co-segregate with the disease and was therefore not included in the HGMD database. Unlike the findings of Albulym et al., in this study, proband 2 exhibited typical symptoms of CMT2Z. Analysis of WES data from proband 2 revealed that only the p.R283H variant of the *MORC2* gene was closely related and co-segregated with the disease within the family. Furthermore, subsequent bioinformatic and functional studies indicated that this variant could significantly impact the protein’s structure and function. Therefore, we believe that this variant is the genetic cause of the disease in family 2.

*In vivo* studies show that knockout of the *Morc2a* gene (the mouse ortholog of human *MORC2*) results in embryonic lethality. Lee et al. reported that *Morc2a* p.S87L mutant mice develop both peripheral and central neuropathies. In their research, no homozygous mice were obtained. *Morc2a* S87L/+ mice exhibit peripheral motor and sensory neuropathy, locomotor dysfunction, skeletal muscle weakness, axonal neuropathy, cerebellar ataxia, and motor neuron degeneration. Notably, *Morc2a* mRNA and *Morc2a* protein expression in the quadriceps muscle and cerebellum were consistently decreased in *Morc2a* S87L/+ mice compared to wild-type mice (29). Similarly, our site-mutation functional study showed that the mutation (c.34G > C) of *MORC2* altered the half-life and stability of *MORC2* protein. Moreover, the mutation (c.848G > A) of *MORC2* affected *MORC2* protein expression at the transcriptional level. We hypothesize that these may partially explain the patients’ condition. However, further experiments are required for verification.

In conclusion, this study reports a novel *MORC2* variant (p.A12P) and validates a rare known *MORC2* variant (p.R283H) in Chinese families with CMT2Z. Functional analyses reveal two distinct pathogenic mechanisms: protein instability for p.A12P and transcriptional reduction for p.R283H. These findings expand the *MORC2* mutation spectrum, provide novel insights into its molecular pathogenesis, and reinforce the importance of genetic testing and counseling for CMT2Z families, particularly in diverse ethnic populations.

## Data Availability

The raw sequencing data generated in this study have been deposited in the GSA-Human database at the China National Center for Bioinformation (CNCB) and are publicly accessible at https://ngdc.cncb.ac.cn/gsa-human/browse/HRA016211. The novel variant c.34G>C;p.A12P has been submitted to ClinVar and assigned the accession number SCV007637056.
