# Supplementary material for: Novel MORC2 variants in Charcot–Marie–Tooth disease type 2Z: genetic and functional insights
Source: Front Med (Lausanne). 2026 Aug 12;13:1864944. doi: 10.3389/fmed.2026.1864944 (PMC13506280; doi:10.3389/fmed.2026.1864944)
Supplement: Supplementary file 1 [file Data_Sheet_1.DOCX]

Novel MORC2 Variants in Charcot-Marie-Tooth Disease Type 2Z: Genetic and Functional Insights

Running Title: *MORC2* variants in CMT disease

Rong Tao^1#^, Ten Zhang^2#^, Fang-Mei Luo^3^, Yihui Liu ^2^, Liangliang Fan^3^, Xuan Wu^2*^, Yuxing Liu^4,5*^

1. Center for Medical Research, Pingshan District Central Hospital of Shenzhen, Guangdong, China;

2. Department of Neurology, Affiliated Hospital of Yangzhou University, Yangzhou, China;

3. Department of Cell Biology, the School of Life Sciences, Central South University, Changsha, China;

4. Laboratory Center, Guangxi Key Laboratory of Metabolic Reprogramming and In-telligent Medical Engineering for Chronic Diseases, the Second Affiliated Hospital of Guilin Medical University, Guilin, China;

5. Laboratory Center, Guangxi Health Commission Key Laboratory of Glucose and Lipid Metabolism Disorders, the Second Affiliated Hospital of Guilin Medical University, Guilin, China.

#These authors contributed equally to this work

* Correspondence:

Xuan Wu, Ph.D

Email: [15861364610@126.com](mailto:15861364610@126.com)

Department of Neurology, Affiliated Hospital of Yangzhou University, 45 Taizhou Road, Yangzhou City, Yangzhou, Jiangsu, 225001, China;

and

Yuxing Liu, Ph.D

Email: [1091158014@qq.com](mailto:1091158014@qq.com)

Central Laboratory, The Second Affiliated Hospital of Guilin Medical University, Guilin, China.

**Figure S1.** Sanger DNA sequencing chromatogram demonstrates the (A) c.34G>C-*MORC2* and (B) c.848G>A;p.R283H missense variants were engineered into the pcDNA3.1-*MORC2*-flag vector.

Table S1. PCR primers used in this study

| Gene | Forward primer | Reverse primer | Experiment |
| --- | --- | --- | --- |
| *RNF220* | CAGAGGATCGGAATGACAGATG | CAGACAACAGGAGGGACTTT | PCR |
| *LMAN2L* | GGGCTGGGAGTATTTGTAGAC | CCAGGAAGGTGTCGTAATGAA | PCR |
| *BDP1* | GTCTAGTGTCAGTGTTCCTTCAG | GGTGGCCTCTGACTTTCATT | PCR |
| *MYO6* | CGTCCTGCACCTTGGAAATA | GTTTGCTTGCTCCACTTTCAG | PCR |
| *PRAG1* | GTGTGAACAGCTCACCTTACT | ACAGCGAGAATTGCCATCA | PCR |
| *DCHS1* | AGACTCAGGACCCAATGGA | AGGTCAAGCACTGCAACA | PCR |
| *ITGA11* | GCACTCAAGTACAAATCCATGAAA | TAAAGAAGCCGAGCTTCCAC | PCR |
| *TOX3* | AGCATCTCCAGCAGCAAATTA | CTGACCTGCGATAATACTTGAGTC | PCR |
| *LRP3* | CAGTGGTCTGTGCTACACG | CACCAGGCTGCCAATGA | PCR |
| *MORC2*-c.34 | GAAGGGCTGGAGACCTTATTG | ACATTTCACTCCAGTGGGATAC | PCR |
| *SH3KBP1* | GTGGAAACTCTTTGCTGTCTTC | ATTCCAGTCTTCCCGTTGAG | PCR |
| *KIAA1217* | CCCGACCACATCATTGCATATC | CGAGGTCTACAAGGCTGGATAA | PCR |
| *PAK6* | TCCAGAAGTTGTCAGTCATCAG | TTGGCAGCCTTCATTCCA | PCR |
| *B9D1* | TTGTCCCAGAATCTACGTCTAAA | CCAACACACCCTGTGTATCA | PCR |
| *OSBPL1A* | AACACAGAAGCGGAGCAA | CGTGTCTCCCATGTCATTCA | PCR |
| *ROMO1* | GGGTCCCTACGGACAGT | GGCCATGAATGTGCCAAAG | PCR |
| *MORC2*-c.848 | TGCTGAATTGAGCTTGGATTTG | GAGTTGTGGACTCAGCCTTT | PCR |
| *DNAH11* | TTCCATTCCACTAACCGAAGG | GACTTTCAGGTCCATTCCATACT | PCR |
| *PAG1* | TGCAGCATTACGAGGAAGTC | GCTCTTTCGAGGCAGAAGTAG | PCR |
| *MORC2-Flag* | GAGCTGGACGCCTACATT | AGTCCTTATCATCGTCGTCTTT | qPCR |
